# Global Interest in Telehealth During COVID-19 Pandemic: An Analysis of Google Trends™

**DOI:** 10.7759/cureus.10487

**Published:** 2020-09-16

**Authors:** Shajeea Arshad Ali, Taha Bin Arif, Hira Maab, Mariam Baloch, Sana Manazir, Fatima Jawed, Rohan Kumar Ochani

**Affiliations:** 1 Internal Medicine, Dow University of Health Sciences, Karachi, PAK

**Keywords:** coronavirus disease 2019 (covid-19), telemedicine, telehealth, google trends, pandemic, infodemiology, global health, public health surveillance

## Abstract

Background

Since the outbreak, healthcare systems across the globe are overcrowded with coronavirus disease (COVID-19) patients. To sustain the response towards the pandemic, many hospitals have adapted to virtual healthcare and telemedicine. Google™ has become the most widely used search engine over the years. Google Trends™ can be used to depict the public interest over a certain topic. The output of the Google Trends™ is displayed as relative search volume (RSV) which is the proportionate search volume regarding a specific topic comparative to the total search volume in a specific time and region. The primary aim of this study was to evaluate the relationship between the daily reported number of new COVID-19 cases and deaths and the corresponding changes in Google Trends™ RSV of telehealth over six months.

Methods

A retrospective study was conducted from January 21, 2020 to July 21, 2020. About 17 countries that reported the total number of cases greater than 200,000 in the situation report of July 21, 2020 were selected to be a part of this study. The daily reported new cases and deaths globally and of the selected countries were extracted from the World Health Organization (WHO) situation reports. The combination of keywords used for obtaining the RSV data through Google Trends™ was “telehealth”, “telemedicine”, “mHealth”, and “eHealth”. These words were used with the “+” feature of Google Trends™ with “1/21/2020 to 7/21/2020” as time range, “all categories” for the category, and “web search” for the type of search. The worldwide RSV as well as the RSVs of the selected countries were obtained from the Google Trends™ website. Spearman’s correlation coefficient (ρ) was used to determine the strength of the relationship between new cases or deaths and RSVs related to telehealth.

Results

A positive fair correlation was established between the global interest in telehealth and the new cases (ρ=0.307, p-value<0.001) and deaths (ρ=0.469, p-value<0.001) reported worldwide. The United States of America (USA), India, and Bangladesh were found to have a positive fair correlation between the public interest regarding telehealth and the emerging new COVID-19 cases and deaths. The United Kingdom (UK) and Italy demonstrated a positive poor correlation between the rising new cases or deaths and RSV. Similar statistics were noted for the daily new cases of Chile. For Turkey, a positive fair correlation between new deaths and RSV while a positive poor correlation between new cases and RSV was observed. No significant correlation was observed for the rest of the selected countries.

Conclusion

This study highlights the steadily rising public interest in telehealth during the COVID-19 pandemic. Telemedicine can provide the necessary remote consultation and healthcare for patients in the current situation. However, previous studies have shown that the majority of the countries are inadequately equipped for the digitization of the healthcare system. Therefore, it has become necessary to incorporate telemedicine into the healthcare system to combat any possible pandemic in the future.

## Introduction

Coronavirus disease 2019 (COVID-19) is a highly transmissible disease having a case fatality rate higher than one percent [1]. The existing healthcare systems and policies have been modifying to cope with the demands since the outbreak [1,2]. Digitization of the healthcare system can aid in the management of the pandemic, mitigating the disruption of societal systems [1]. The World Health Organization (WHO) has recognized digital healthcare for having the potential to tackle this global threat and contribute to supporting the response towards the pandemic [2]. The current pandemic is distinctive from its predecessors in the number of individuals infected, transmission rates, and the range of clinical severity. It is the first pandemic to occur in the digital era with sophisticated, timely aged, and mature digital health solutions that can play a considerable role in the surveillance and management of this crisis [2]. Using real-time internet data is one way in this direction of using modern methods for disease surveillance. On the concept of information epidemiology, the term ‘infodemiology’ was coined by Gunther Eysenbach. Infodemiology involves the dissemination of information in an electronic medium, particularly the internet, or in a population with the fundamental objective to inform public healthcare policy [3]. The use of population health tools and technologies like the internet was mentioned by Eysenbach during the severe acute respiratory syndrome (SARS) epidemic. Online search engines harvest a huge quantity of real-time data from the public and this has led to an interest in utilizing this data for public health use during possible infectious disease outbreaks [4].

Google Trends™ is one such example of Big Data information surveillance tools used for the analysis and interpretation of temporal and geographical trends of online search terms using the Google™ search engine [4]. Analyzing relative internet search volumes (RSV) with Google Trends™ provides information on the magnitude of public interest on a certain topic [3]. As these searches are time stamped, the timing of these searches can be correlated to a certain public event or confirmed disease spread and can be used to predict the dissemination of disease from these events [4]. For example, in Indonesia, Google Trends™ data had a linear time series pattern and it statistically correlated with the annual official dengue report [5]. Considering the COVID-19 pandemic, Google Trends™ was used to demonstrate a statistical correlation between online interest and COVID-19 cases and deaths in Europe [6].

Telemedicine, on the other hand, can be regarded as applying technology for conducting clinical medicine from a distance and establishing a connection between the physicians and patients in multiple settings [7]. Telemedicine is a component of telehealth, which itself is a constituent of electronic health (e-health), along with mobile health (m-health) and electronic medical record (EMR) [8]. With stay-at-home orders in place as numerous countries underwent strict lockdowns and suspension of non-essential in-person clinic encounters, it was increasingly imperative to shift services virtually for the provision of uninterrupted clinical facilities. Consultations through telemedicine underwent an increase of 683% during the COVID-19 pandemic in New York City [9]. Some patients may even have delayed emergency care during the early days of COVID-19 [10]. In the United States of America (USA), the Centers for Disease Control and Prevention (CDC) estimated a 42% decline in the emergency department visits compared to the last year. In case of serious conditions, this might lead to complications or even deaths [10]. Telemedicine, in these circumstances, increases the patient’s access to medical advice facilitating patient-centered medical consultations [8]. By restricting physical human interaction, it can likewise help in confining the spread of severe acute respiratory syndrome coronavirus 2 (SARS-CoV-2).

With the shift in the paradigm of healthcare consultation in the era of COVID-19, it is rational to presume that this change must have gone hand in hand with increased online public interest over the topic, possibly before scheduling a teleconsultation or just due to plain human curiosity. As increased RSV obtained via Google Trends™ is a reflection of increased online public interest, the primary objective of this study was to assess the association between the daily reported number of new COVID-19 cases and deaths and simultaneous changes in RSV of telehealth over six months in different countries across the world.

## Materials and methods

Study tool

Google Trends™ has become a popular tool in analyzing the public’s interest in health topics and predicting human behavior during disease outbreaks [11]. Since Google™ is the most used search engine, it can accurately depict the global interest of any chosen topic [12]. In various countries, official health data are not accessible to the general public which makes the forecasting and preparedness of future outbreaks an arduous and lengthy process. Google Trends™ enables its users to extract the present and archived data of any selected keywords searched via Google™ from 2004 onwards which can be localized over different regions and periods [11]. The selection of appropriate keywords for analyzing Google™ search queries is imperative for obtaining accurate results. For this reason, the “+” feature of Google Trends™ can be utilized which enables the user to include the synonymous or related words of a search term. This creates a combination of keywords that takes into account all possible search requests for a health topic of interest to carry out a detailed analysis [11].

The output from the Google Trends™ is displayed as RSV, which expresses the search volume corresponding to the number of times a keyword has been searched through Google™. It shows a proportionate search volume of the specific keywords about all the other searches conducted within the same time and region. It is displayed on a scale of 0 to 100 where 100 is the peak of a search term for the selected duration and geographic location while 0 indicates an almost negligible number of search requests [3].

Study design

This was a retrospective study conducted to determine the relationship between the new COVID-19 cases and deaths and the public interest towards the field of telehealth. To get a detailed analysis, a six-month time range was selected for the study from January 21, 2020 to July 21, 2020.

Selection criteria

The WHO started publishing daily situation reports for the surveillance of the COVID-19 pandemic on their website from January 21, 2020 [13]. It reported the official count of total confirmed cases and deaths as well as the number of new cases and deaths in each country. Testing for the SARS-CoV-2 has been impeded in numerous countries due to the dearth of health funds and testing equipment [14]. As a result, there has been inaccuracy in the reported number of confirmed cases at a specific time. Hence, we chose to determine the relationship between the RSV obtained from Google Trends™ and the number of new cases and deaths reported per day.

New COVID-19 Cases and Deaths

The countries reporting more than 200,000 confirmed cases of COVID-19 in the situation report of July 21, 2020 were selected to be a part of the study [15]. The number "200,000" was arbitrarily selected to include the maximum regions of the world in our analysis. Based on this criterion, the following countries were included: South Africa, USA, Brazil, Peru, Chile, Mexico, Iran, Pakistan, Saudi Arabia, Russian Federation (Russia), United Kingdom (UK), Spain, Italy, Turkey, Germany, India, and Bangladesh. The data of these selected countries as well as the worldwide data of new cases and deaths were extracted from the situation reports published on the website of WHO from January 21, 2020 to July 21, 2020 [13].

RSV Obtained From Google Trends™

The combination of keywords selected for our study was “telehealth”, “telemedicine”, “eHealth”, and “mHealth”, which were used with the “+” feature of Google Trends™. These words were selected on the basis that they are used interchangeably by the public while searching for the availability of telehealth services in their area. The data of these search terms were downloaded from the Google Trends™ website [16].

The following filters were selected for extracting the data from Google Trends™: “1/21/2020 to 7/21/2020” as time range; “all categories” for the category, and “web search” for the type of search. The parameter “region” was changed in accordance with each of the countries selected to obtain accurate RSVs. The worldwide RSV was downloaded from the site to sketch a global picture of public interest in telehealth during the current pandemic [16].

Statistical analysis

The data extracted from the various sources were entered and analyzed through the Statistical Package for Social Sciences (SPSS) version 24.0 (IBM Corp., Armonk, NY). The RSV data of each country for the search terms from 01/21/2020 to 07/21/2020 were plotted against the new COVID-19 cases and deaths reported from that country. The data were examined graphically through the histogram using the feature of the bell-shaped curve and tested by the Shapiro-Wilk test. As the data were confirmed to be aberrantly distributed, Spearman’s rank-order correlation test was used to analyze the association between the RSV and the number of new COVID-19 cases and deaths globally and in each of the selected countries. The Spearman’s correlation coefficient denoted by ‘ρ’ was used to determine the strength and direction of the relationship between two variables. The value of ρ lies between -1 to +1, where the “+” sign shows a positive correlation, the “-” sign indicates an inverse relation, and “0” demonstrates no correlation between the variables. The value of ρ was interpreted based on the criteria devised by Chan. The value of ρ ≥ 0.8 was used to depict a very strong relation, ρ < 0.8 and ≥ 0.6 for moderately strong relation, ρ < 0.6 and ≥ 0.3 for fair relation, and ρ < 0.3 for a poor relation between the variables [17]. A p-value of < 0.05 was considered statistically significant.

## Results

The means of Google Trends™ RSVs regarding telehealth

Worldwide

The global interest regarding telehealth was assessed during the COVID-19 pandemic. As shown in Table 1, a mean of the global search volume regarding telehealth was calculated to be 30.650 ± 20.619. Based on the study’s selection criteria, countries from Europe, South-East Asia, the Americas, Africa, and Eastern Mediterranean regions of the world were included.

**Table 1:** Means of Google Trends™ RSVs related to telehealth during the COVID-19 pandemic RSV: Relative search volume; COVID-19: Coronavirus disease 2019

Regions	Mean	Standard deviation
Worldwide	30.650	20.619
Iran	10.825	20.426
Pakistan	20.147	20.799
Saudi Arabia	15.361	24.576
India	38.082	24.106
Bangladesh	25.279	25.154
South Africa	16.290	23.165
Russian Federation	17.519	24.438
United Kingdom	4.093	7.301
Spain	23.147	27.736
Italy	25.404	27.385
Turkey	18.530	27.234
Germany	35.869	19.709
United States of America	25.159	20.906
Brazil	25.399	22.962
Peru	9.273	23.018
Chile	9.705	26.499
Mexico	20.923	28.490

Eastern Mediterranean Region

Amongst the three countries selected from the Eastern Mediterranean region, Pakistan was found to have the highest mean of search volume regarding telehealth (20.147 ± 20.799). The RSV of Saudi Arabia was calculated to be 15.361 ± 24.576. Iran had the lowest mean of RSV in this region (10.825 ± 20.426).

Region of South-East Asia

From the region of South-East Asia, both India and Bangladesh were found to have a greater mean of RSVs as compared to the countries from the Eastern Mediterranean region (38.082 ± 24.106 and 25.279 ± 25.154, respectively).

Region of Africa

South Africa was the only country selected from this region. The mean RSV of South Africa was calculated to be 16.290 ± 23.165.

European Region

There were six countries included from the European region. Germany was found to have the highest mean of RSV amongst all the selected countries (35.869 ± 19.709) while the UK was observed to have the lowest mean of RSV (4.093 ± 7.301). Almost similar means of RSVs were noted in Russia and Turkey (17.519 ± 24.438 and 18.530 ± 27.234, respectively) indicating a comparable public interest regarding telehealth between both countries. Similar figures were observed in the means of RSVs of Spain and Italy (23.147 ± 27.736 and 25.404 ± 27.385, respectively).

Region of the Americas

Five countries were selected from the region of the Americas. Brazil was found to have the highest mean of RSV (25.399 ± 22.962) followed by the USA (25.159 ± 20.906). Similar means of RSV were observed in Peru and Chile (9.273 ± 23.018 and 9.705 ± 26.499, respectively) indicating a comparable public interest towards telehealth. Mexico was noted to have a mean RSV of 20.923 ± 28.490.

Google Trends™ RSVs for telehealth plotted against the emerging COVID-19 cases and deaths

Worldwide

**Figure 1:**
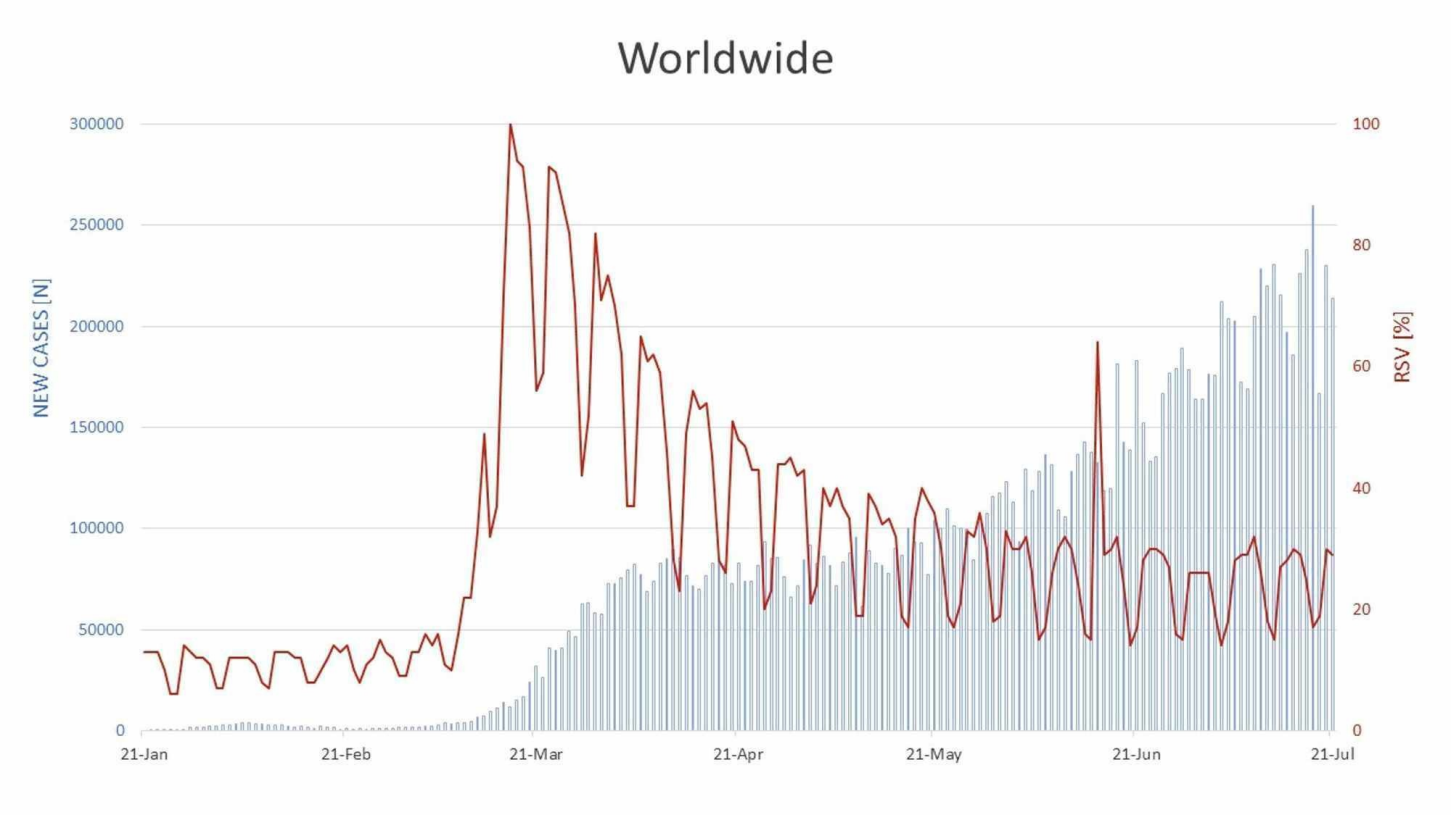
RSV of global public interest regarding telehealth plotted against new cases of COVID-19 reported per day RSV: Relative search volume; COVID-19: Coronavirus disease 2019

The global interest in telehealth during the COVID-19 pandemic was plotted against the new cases reported worldwide per day. As illustrated in Figure 1, the selected time range was January 21, 2020 to July 21, 2020. During January and February, it was observed that the new COVID-19 cases rose with a flat trajectory, with the highest number of cases spiking on February 5, 2020 (N=3925). On March 18, 2020, the reported cases per day crossed the 15,000 new cases per day record. Remarkably, the maximum RSV (RSV=100) for our search terms was recorded on March 17, 2020. From mid-March onwards, the emerging cases took a steady rising slope, with an overall increasing graph trend till the end of six months. During this span, two parallel tall peaks for the new cases were seen on June 18, 2020 and June 21, 2020, respectively. The highest number of cases was reported on July 18, 2020 (N=259,848). Interestingly, the RSV slope illustrated a constant decreasing trend after March 17, 2020 during which it peaked on one occasion, i.e., on June 15, 2020 (RSV=64).

**Figure 2:**
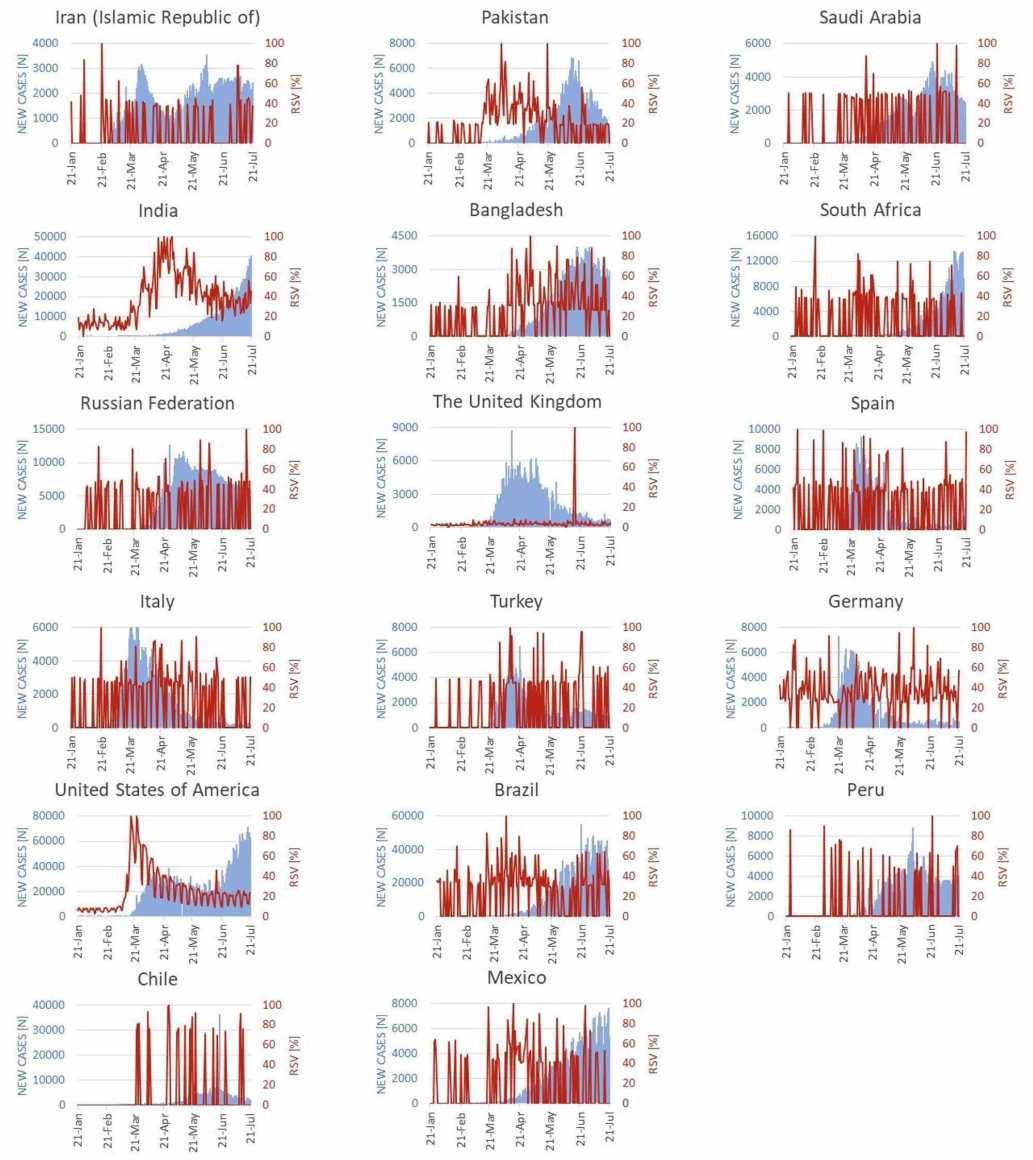
RSV of public interest regarding telehealth plotted against new cases of COVID-19 reported per day RSV: Relative search volume; COVID-19: Coronavirus disease 2019

The public interest in telehealth during the pandemic was assessed in seventeen countries as shown in Figure 2. The Google Trends™ RSVs of the selected countries were plotted against the new COVID-19 cases reported per day from January 21, 2020 to July 21, 2020.

Eastern Mediterranean Region

For Iran, the maximum value of RSV (RSV=100) was observed when there were only three new COVID-19 cases reported in the country. The Iranian Google Trends™ RSV remained zero during most of the days, depicting a lack of interest on the subject. Unlike Iran, where the first peak for new cases emerged in February, the peaks for new cases in Pakistan and Saudi Arabia appeared much later. In Pakistan, the RSV trajectory was seen to rise from March till May. This was the period when the new cases in the country were steeply rising. The RSV peaks (RSV=100) were recorded twice during this interval, on April 4, 2020 and May 20, 2020. The highest number of new cases in Saudi Arabia were reported on June 19, 2020 (N=4757). The maximum RSV (RSV=100) was recorded four days later. Another peak of RSV (RSV=98) was observed in Saudi Arabia almost a month later when the number of emerging cases were steadily declining.

Region of South-East Asia

The first case of COVID-19 in India was reported on January 30, 2020. Subsequently, a single case was reported on February 2, 2020 and February 3, 2020. The next new case emerged one month later after which a steady ascent was observed in the number of new cases. India’s RSV trend fluctuated irrespective of the increasing new cases. Multiple RSV peaks (RSV=100) were observed during the last two weeks of April 2020.

On July 3, 2020, the maximum number of new cases was reported in Bangladesh (N=4019). The Google Trends™ RSV was estimated to be 88, which is one of the few occasions when RSV peaks were observed for Bangladesh.

Region of Africa

In South Africa, the maximum number of new cases emerged in July, with the highest number of new cases reported on July 10, 2020. However, the RSV trend of South Africa in July remained mundane with the maximum RSV reported on July 8, 2020 (RSV=71), two days before the highest number of recorded cases.

European Region

Among the six European countries evaluated in our study, four countries followed a similar pattern in their RSV trends. These included Russia, Spain, Italy, and Turkey. The RSV fluctuated between the values of 0 to 50 in these countries for most of the assessed period. The highest number of new cases for Russia, the UK, Spain, and Turkey were reported in April. In Italy and Germany, the summits were seen in March.

Russia was observed to have the highest peak of RSV (RSV=100) in July when there was a gradually declining curve of COVID-19 cases reported per day. The RSV trend for the UK remains distinct as it persistently maintained minimal figures and rose to its maximum (RSV=100) only once during June. In Spain, the RSV peak (RSV=93) was observed three days after the highest number of cases (N=9222) were reported on April 1, 2020. A similar trend was observed in Italy. The maximum number of new cases (N=6557) was reported on March 22, 2020, and a peak of 81 was observed in the RSV five days later.

In Turkey, the highest number of new cases emerged in April. In concordance with this, there were two notable peaks observed in the RSV on April 11, 2020 (RSV=100) and April 13, 2020 (RSV=92). Conversely, in Germany, the number of new cases rose steadily in March. Germany had most of its RSV in between 25 and 55. However, a peak of 92 was observed in March.

Region of the Americas

The Google Trends™ RSV plot for the USA had the greatest resemblance to the worldwide RSV plot. New cases initially culminated from the beginning of March, reaching the peak on April 26, 2020 (N=38509) after which they declined. The number of new cases started rising again by the end of June and followed a positive gradient subsequently. There were two summits observed in the trajectory of RSV (RSV=100) on March 17, 2020 and March 23, 2020 corresponding to the slope of emerging cases. In Brazil, the RSV was mostly observed to be in the range of 30 to 60. The number of new cases started to rise after mid-March in Brazil reaching the highest point on June 21, 2020 (N=54771). A notable peak of RSV (RSV=100) was observed on April 3, 2020.

For Peru, the RSV was estimated to be either 0 or above 45. The reported cases per day began to rise gradually in April reaching a peak on June 2, 2020 (N=8805). The plotted line of RSV reached the highest point (RSV=100) on June 23, 2020. Like Peru, the plotted line of RSV for Chile either remained 0 or increased above 70. A prominent summit was observed on June 18, 2020 (N=36179) in the trajectory of gradually rising cases of COVID-19. In Mexico, the emerging new cases of COVID-19 gradually started rising from April and followed a positive gradient until July 21, 2020. Two prominent peaks were observed in the RSV on April 14, 2020 (RSV=100) and June 26, 2020 (RSV=98), respectively.

Spearman’s correlation analysis between Google Trends™ RSVs and new COVID-19 cases and deaths

Worldwide

The global search volumes regarding telehealth and the RSVs of the selected seventeen countries indicating the public’s interest in this topic were gathered. These RSVs were plotted against the new COVID-19 cases and deaths reported per day. Spearman’s correlation test was applied to determine the strength of association between the RSVs regarding telehealth and the new COVID-19 cases and deaths. As shown in Table 2, a positive fair relation was found between the new cases of COVID-19 reported worldwide per day with the global interest in telehealth (ρ = 0.307, p-value < 0.001). A similar relation was also established with the number of new deaths reported worldwide (ρ = 0.469, p-value < 0.001).

**Table 2:** Spearman’s correlation of RSVs and new COVID-19 cases and deaths ^a^ indicates p-value < 0.05 which was considered statistically significant; ^b ^indicates positive fair correlation; ^c^ indicates positive poor correlation RSV: Relative search volume; COVID-19: Coronavirus disease 2019

Region	New COVID-19 cases and deaths	Spearman’s correlation coefficient (ρ)	P-value
Worldwide	New cases	0.307^b^	<0.001^a^
	New deaths	0.469^b^	<0.001^a^
Iran	New cases	0.031	0.681
	New deaths	0.079	0.285
Pakistan	New cases	0.079	0.289
	New deaths	0.053	0.479
Saudi Arabia	New cases	0.122	0.100
	New deaths	0.083	0.265
India	New cases	0.473^b^	<0.001^a^
	New deaths	0.495^b^	<0.001^a^
Bangladesh	New cases	0.436^b^	<0.001^a^
	New deaths	0.439^b^	<0.001^a^
South Africa	New cases	-0.002	0.978
	New deaths	-0.004	0.955
Russian Federation	New cases	0.054	0.471
	New deaths	0.063	0.399
United Kingdom	New cases	0.266^c^	<0.001^a^
	New deaths	0.243^c^	0.001^a^
Spain	New cases	0.070	0.345
	New deaths	0.018	0.810
Italy	New cases	0.252^c^	0.001^a^
	New deaths	0.239^c^	0.001^a^
Turkey	New cases	0.267^c^	<0.001^a^
	New deaths	0.307^b^	<0.001^a^
Germany	New cases	-0.072	0.333
	New deaths	0.023	0.757
United States of America	New cases	0.429^b^	<0.001^a^
	New deaths	0.513^b^	<0.001^a^
Brazil	New cases	0.052	0.484
	New deaths	0.031	0.672
Peru	New cases	0.133	0.073
	New deaths	0.098	0.186
Chile	New cases	0.152^c^	0.040^a^
	New deaths	0.127	0.087
Mexico	New cases	0.049	0.514
	New deaths	0.075	0.316

Eastern Mediterranean Region

None of the countries selected from the Eastern Mediterranean (Iran, Pakistan, and Saudi Arabia) proved to have a significant relationship between rising new cases or deaths of COVID-19 and public interest regarding telemedicine (p-value>0.05).

Region of South-East Asia

India was found to have a positive fair relation between new COVID-19 cases (ρ = 0.473) or deaths (ρ = 0.495) and RSV (p-value < 0.001). Likewise, a positive fair association was proved between the RSV and new cases (ρ = 0.436) as well as new deaths (ρ = 0.439) reported per day in Bangladesh (p-value < 0.001).

Region of Africa

No significant correlation was found between new cases or deaths and RSV for telehealth in South Africa (p-value>0.05).

European Region

European region had the maximum number of countries afflicted from the expeditious local transmission of COVID-19. However, only 50% of the selected countries were proved to have a significant relationship between newly reported cases or deaths and RSV indicating public interest in telehealth. These included the UK, Italy, and Turkey. Turkey was found to have a positive fair correlation between RSV and new deaths (ρ = 0.307). However, a positive poor association was confirmed between new cases and RSV (ρ = 0.267). The UK (ρ for new cases = 0.266, ρ for new deaths = 0.243) and Italy (ρ for new cases = 0.252, ρ for new deaths = 0.239) were both found to have a positive poor correlation between the RSVs and rising new COVID-19 cases or deaths.

Region of the Americas

From the region of the Americas, Chile was found to have a positive poor association between the RSV and emerging new cases (ρ = 0.152, p-value = 0.040). Additionally, a positive fair correlation was found between the RSV and new cases or deaths reported per day (ρ for new cases = 0.429, ρ for new deaths = 0.513) in the USA. There was no significant association between RSV and new COVID-19 cases or deaths in Brazil, Peru, and Mexico.

## Discussion

One of the advancements in the twenty-first century is the use of telehealth technology that enhances access to care, improves the quality of care delivery, and ameliorates the doctor-patient engagement and satisfaction [18]. With the rapid evolution in portable electronic devices, extensive work on e-Health has occurred in the high to middle-income countries [19,20]. Telehealth related activity has been operational for the last 20 years. A recent global survey by the WHO indicates an accelerating trend towards e-Health with 58% of countries having an e-Health strategy [21]. About 57% of the member states responded affirmatively to the presence of national telehealth policy. However, only a few telehealth programmes were being operated internationally [21].

Pandemics and disasters pose significant challenges in the adequate delivery of health care. Amidst the catastrophe by COVID-19 across the world, a significant consideration to telehealth could play a critical role in the provision of global healthcare and can become a necessity for the general population. Being a communicable disease, the risk of transmission of COVID-19 can be decreased by remote assessment of patients [22]. Moreover, individuals who are not infected with COVID-19 but are at higher risk of getting infected (e.g., old people with comorbidities) can easily adapt to the system without the risk of exposure to infection by visiting hospitals. Patients living in isolation or quarantine can develop a substantial interaction with their healthcare providers on mobile phones, webcams, or portable tablets. The efficacy of telehealth in the COVID-19 pandemic can be determined by the appropriate integration in the usual healthcare system [22].

Using Google Trends™, our study found a significant worldwide correlation between RSV for telemedicine and new COVID-19 cases and deaths. Although the attention to COVID-19 increased promptly even before the actual peak outbreak [3], the importance of telemedicine can be seen to rise during the excessive periods of distress secondary to the culminating number of COVID-19 cases. This strongly suggests that RSV can be used to monitor the response of the public towards the local and global outbreaks. Various search engines and social media platforms are a major source of information regarding real-time data on telehealth services. With the introduction of terms like “social distancing” and “medical distancing”, the population has prioritized a remote approach for seeking healthcare. The telehealth services are laid on a virtual network under the guidance of the central clinic. This network contains physical locations of central and remote clinics, private healthcare centers, and rehabilitation setups. Thus, the telehealth is a paramount strategy to keep the people discrete or the caregivers discrete from patients and other providers [23].

Since the past decade, the influence of web-based search has escalated continuously [24]. The Google Trends™ of users’ searches have been widely analyzed and used in the context of health problems and their remedies. An example of a drastic escalation in public interest in telemedicine during the COVID-19 pandemic is Ethiopia, depicting the highest interest rate of telemedicine-related Google™ searches. In January 2020, the keyword “telemedicine” reached an interest rate of 15, which slightly increased to 17 in February. With the implementation of lockdown in March, the popularity score of this keyword drastically increased to 97, demonstrating a 546% growth rate. This interest peaked in April, reaching a score of 100 and representing a surge of 525% compared to the statistics in January [25]. Hong et al. found an increase in the Google Trends™ RSV of telemedicine with the rising COVID-19 cases in the United States. However, the magnitude of population interest did not correlate with the number of telehealth facilities. These findings conclude that the growing population demand cannot be met by the existing telehealth capacity [12].

Although previously reported data has shown the advantages of telemedicine in terms of reduced traveling and recognizable convenience, people are more considerate about the equivalence of telemedicine to the physician visits [26]. A lower literacy rate, lack of funding and adequate resources, and paucity in the definite existing infrastructure are potent barriers to the implementation of telehealth [21]. The obstacles to the development of telemedicine culture include technology and non-technology issues. The non-technology barriers include inadequate supervision or legislation, immediate and widespread implementation breakdown, unavailability of technical expertise and support, and resistance to evaluation protocol and changing habits [21,27]. A drastic negative impact on the economic growth of many countries has been observed since the outbreak of COVID-19. A six percent reduction in the global economic growth rate in 2020 has been estimated. Experts say that these diminutions will be partially compensated if there is no second wave of infection [28].

Our study is subject to certain limitations. First, the sole use of Google Trends™ does not incorporate the entire internet search traffic. Approximately 72% of the search is conducted on Google™ [29]. Our analysis does not include the remaining search activity conducted on other search engines. Second, the precision of RSV in measuring the public interest has not been approved although it offers a novel method of estimating public interest [29,30]. Third, different countries have several platforms, languages, and communication channels. Hence, our analysis may not comprise all search terms in native languages used by the public of selected countries due to the deficiency of a standardized criterion. Fourth, the analysis is subject to inherent bias due to the anonymity of data in Google Trends™. Google Trends™ generates data of literate, technology competent population with access to internet facilities (specifically Google™). Therefore, we could not determine the segments of the population that may be underrepresented or excluded from the analysis.

A multifaceted approach can be used to overcome the barriers in the implementation of telemedicine services. The substantial strategies include the introduction of a trained workforce or expertise in using e-Health solutions, adequate governance, and proper funding. Creation and surveillance of standardized, legal, and technical infrastructure with a detailed plan of action and vision is a fundamental building block of national e-Health strategy [21]. An increase in the online interest of the population towards telehealth does not estimate the actual count of people seeking teleconsultation. Therefore, a national-level health survey should be conducted to determine the knowledge, prevalence, and predictors of teleconsultation. This may include the feedback of clinicians and patients after an e-Health appointment. The ambiguity regarding the provision and utilization of telehealth services should be cleared out. A systematic approach towards telehealth through e-learning should be established. This may include easy access to instructions providing multilingual knowledge support to the health staff and citizens through campaigns on electronic media. With regard to the pandemic, the tracking, amenity, and precision of the regional coverage monitoring network can be improved by periodic use of electronic health records. In addition, cost-effective solutions for providing e-Health should be prioritized. The technology for mobile communications can be supplied at cheaper rates compared to providing in-person assistance. The introduction of mobile phones, patient monitoring devices, personal digital assistants, and wireless devices for medical and public health practice has revolutionized the concept of telehealth into mobile health (also known as m-Health) [21]. It can be used to provide better healthcare services in the regions having a fragile infrastructure to sustain the cyberspace or other technologies. In conclusion, governments should ensure the public dissemination of lessons and benefits of implementing telemedicine, release a well-governed e-Health policy, and build sponsored e-Health programmes for propagation and prosperity of telemedicine in challenging times like the COVID-19 pandemic.

## Conclusions

Telemedicine can perhaps help by allowing sick people to get high-quality supportive care without any exposure to severely ill patients. Noticing the trend of global interest in telemedicine during the peak of outbreaks can help us devise better healthcare policies and strategies. Using Google Trends™, our study found a significant escalation of worldwide interest towards telehealth during the current pandemic. However, previous surveys have shown a lack of adequate infrastructure and surveillance of available telemedicine networks. Digitization of health services can also be the novel rising field in the post-COVID-19 period. This strongly highlights the necessity of telemedicine and its amalgamation into the healthcare system for surviving any plausible pandemic in the future.
